# Cost-effectiveness of immediate septoplasty versus medical management with the option for delayed septoplasty for nasal airways obstruction: a multicentre, open-label, randomised controlled trial

**DOI:** 10.1136/bmjopen-2025-107402

**Published:** 2026-07-06

**Authors:** Tara Homer, Sean Carrie, Tony Fouweather, Dawn Teare, Nichola Waugh, James O’Hara, Janet A Wilson, Laura Ternent

**Affiliations:** 1 Population Health Sciences Institute, Faculty of Medical Sciences, Newcastle University, Newcastle upon Tyne, UK; 2 Department of Ear, Nose and Throat, Newcastle Upon Tyne Hospitals NHS Foundation Trust, Freeman Hospital, Newcastle upon Tyne, UK; 3 Newcastle Clinical Trials Unit, Newcastle University, Newcastle upon Tyne, UK

**Keywords:** Health economics, OTOLARYNGOLOGY, SURGERY

## Abstract

**Objectives:**

To estimate the cost-effectiveness of immediate septoplasty compared with 6 months of medical management with the option for delayed septoplasty in individuals with nasal obstruction associated with septal deviation.

**Design:**

Economic evaluation alongside a multicentre, open label, randomised controlled trial.

**Setting:**

17 otolaryngology clinics in the UK, recruiting from January 2018 to December 2019.

**Participants:**

Adults aged≥18 years with symptoms of nasal obstruction associated with septal deviation with at least moderate symptoms of nasal obstruction (score>30 on the Nasal Obstruction and Symptom Evaluation scale).

**Interventions:**

Participants were randomised to receive either septoplasty within 12 weeks of recruitment or 6 months of medical management (nasal steroid and saline spray) with the option for delayed septoplasty.

**Primary and secondary outcome measures:**

Incremental cost per quality-adjusted life year (QALY) gained at 12 months. A UK National Health Service perspective was adopted, and surgery costs were estimated using a tariff and micro-costing. QALYs were estimated based on responses to the Short Form-36 (SF-36). Seemingly unrelated regression was used to estimate incremental costs and QALYs. A model-based analysis was used to extrapolate costs and effects to 24 months. Sensitivity analyses were used to illustrate uncertainty.

**Results:**

In the within-trial analysis, immediate septoplasty was on average more costly (mean difference (95% CI) £1193 (£1018 to £1368)) and more effective (mean difference (95% CI) 0.044 QALYs (0.03 to 0.06)) when compared with 6 months of medical management with the option for deferred septoplasty. Immediate septoplasty had an incremental cost per QALY gained of £27 114 in the base case analysis, which decreased to £16 682 when micro-costing was used to estimate surgery costs. Immediate septoplasty had a 15% and 78% probability of being considered cost-effective at a £20 000 threshold for an additional QALY, respectively. In the model-based analysis, immediate septoplasty remained more costly and more effective than 6 months of medical management with the option for deferred septoplasty but had a 99% probability of being considered cost-effective at a £20 000 threshold for an additional QALY.

**Conclusions:**

Over a 24-month time horizon, immediate septoplasty would be cost-effective in the management of deviated nasal septum.

**Study registration:**

ISRCTN16168569.

**STRENGTHS AND LIMITATIONS OF THIS STUDY**

- Data used to inform the economic analysis were collected prospectively as part of a large randomised controlled trial; hence, the results are robust.
- Sophisticated statistical methods were used to estimate the differences in costs and effects and to account for missing data.
- Two different costing methods were used to estimate the costs associated with septoplasty.
- Data to inform the micro-costing of septoplasty were only collected from those randomised to septoplasty; hence, variations in surgical costs could not be captured in the bootstrapping.
- The 12-month trial follow-up, while pragmatic, was arguably too short for the full consideration of costs and benefits associated with a surgical intervention to be considered.

## Introduction

A deviated nasal septum, which could potentially occur because of injury, often presents as a narrowing on one side of the nose which can obstruct airflow. Symptoms of a deviated septum include a blocked nose, snoring and sleep disturbance. Septoplasty is an operation that straightens the nasal septum for individuals with a deviated nasal septum and aims to reduce these symptoms.^1^ However, there is limited evidence about the effectiveness of septoplasty and which individuals would be most likely to benefit from this management strategy. Additionally, as with all surgical procedures, there are risks of complications that also need to be considered alongside the potential costs to individuals who are undergoing the surgery. Most individuals undergoing septoplasty need to take at least 5 days off paid work or usual activities, and some may not see an improvement in symptoms after the surgery.^2^ The initial management strategy for a deviated septum is often a trial of medical treatment in the form of a daily steroid spray. Septoplasty is usually offered to those patients who either fail to improve on such treatment or those who express a preference for surgery. Similar to septoplasty, there is no clear guidance available on how these sprays should be prescribed; however, medical management is often the preferred choice in commissioning guidelines.^3^ Despite the potential uncertainty associated with recommending septoplasty as the management strategy for a deviated nasal septum, over 8500 septoplasty operations were carried out on adults in the UK National Health Service (NHS) in 2023/2024, costing over £23 million.^4^

Given that there is currently no good evidence about septoplasty or its alternatives, or about who might benefit most from treatment to inform national and international guidance and practice in septoplasty, the Nasal AIRway Obstruction Study (NAIROS) was funded by the National Institute of Health Research Health Technology Assessment. NAIROS is a funded multicentre, randomised controlled trial (RCT) with a qualitative process and economic evaluation. The aim of NAIROS was to provide guidance on the use of septoplasty by determining the clinical- and cost-effectiveness of surgical (immediate septoplasty) compared with medical treatment (6 months of isotonic and steroid nasal sprays with the option for delayed septoplasty) in the management of nasal obstruction associated with a deviated nasal septum. This paper reports the results of the economic evaluation, which was undertaken alongside the clinical trial. To our knowledge, this is the second economic evaluation investigating the cost-effectiveness of septoplasty and the first to evaluate the cost-effectiveness of septoplasty compared with 6 months of medical management (nasal sprays) with the option for delayed septoplasty in the management of a deviated septum. The trial clinical outcomes are presented elsewhere.^5^

## Methods

The economic evaluation reported adheres to the Consolidated Health Economic Evaluation Reporting Standards.^6^ The economic evaluation consisted of a within-trial analysis which estimated the incremental cost per quality-adjusted life year (QALY) gained at 12 months and a model-based analysis which estimated the incremental cost per QALY gained at 24 months. All analyses were prespecified in a health economics analysis plan.

The NAIROS trial was a multicentre, non-adaptive, open-label RCT conducted across 17 otolaryngology clinics in England, Scotland and Wales. Participants were stratified by sex and baseline severity and were randomised 1:1 to the two management strategies. The primary outcome, which estimated improvements in patient-reported outcomes, was estimated at 6 months using the Sino-Nasal Outcome Test (SNOT-22). Further details of the trial design, inclusion criteria and clinical effectiveness outcomes, including the primary outcome which was estimated at 6 months, are presented elsewhere.^5 7^ Costs and effects incurred in the economic model after 12 months were discounted at 3.5%.^8^ Data analysis was conducted using STATA software.^9^

### Estimation of costs

UK NHS and personal social service perspectives were adopted for the collection of costs, which are presented as Great British pounds (GBP) (£) in 2020. Costs were based on the interventions (surgery and nasal sprays), the use of healthcare services and concomitant medications.

In the basecase analysis, the cost of surgery was assumed to be the day-case tariff for septoplasty (CA11A) reported in the National Schedule of Reference Costs,^10^ and this cost was assigned to all participants who received the surgical intervention. A sensitivity analysis adopted a micro-costing technique using information collected in the electronic Case Report Form (eCRF), which was completed only for those randomised to septoplasty who received the surgical intervention. The eCRF collected information on the duration of the surgery, the grade of the senior surgeon and senior anaesthetist present during the surgery, discharge medication and the duration of the participants’ admission. The unit costs of each staff member present were based on national pay scales and multiplied by the duration of the surgery to estimate the total staff costs for each septoplasty performed.^11^ For staff costs it was also assumed, based on clinical advice, that a scrub nurse, floor nurse, healthcare assistant, operating department practitioner (ODP) and an anaesthetic specialist registrar would also be present for the duration of the surgery. The costs of consumables and reusables were collected from one of the participating sites (personal communication with Sean Carrie and Graham Stobbs, The Newcastle upon Tyne Hospitals NHS Foundation Trust, 08 April 2021); additional resources were assigned if turbinate reduction was performed (online supplemental table S1). The name, dose, frequency and duration of all discharge medication were reported in the eCRF, and the costs of these medications were collected from routine sources.^12^ An additional cost was assigned if the participant was admitted overnight.^10^ The total cost of surgery, based on the micro-costing estimates, was estimated for each participant randomised to septoplasty who received the surgery. These costs were then aggregated to estimate the average total cost of surgery, which was assigned to all participants who received septoplasty, regardless of their randomised allocation. As part of the trial design, based on patient and public involvement (PPI) during the design stage of the study, participants randomised to medical management had the opportunity to discuss the option of septoplasty after the 6-month primary outcome time point.

Five Sterimar spray canisters and 11 Mometasone nasal spray bottles were provided to all participants randomised to medical management with the option for deferred septoplasty and were to be used daily for 6 months. The cost of these sprays was assumed regardless of compliance, and these costs were collected from routine sources.^12^

A Health Utilisation Questionnaire (HUQ), designed based on previous studies and feedback from the Trial Management Group and PPI representatives, was completed by participants at baseline, 6- and 12-month post-randomisation. The HUQ collected information on primary and secondary healthcare resource use. Primary care consultations could be held face-to-face at participants’ general practice or in their home. Consultations over the telephone, including calls to NHS call centres, were also collected in the HUQ. Secondary care resource use included day and overnight admissions to hospitals, visits to accident and emergency and outpatient consultations. Unit costs of healthcare resources were obtained from routine sources.^10 13^

Additional medications taken by participants to manage their nasal obstruction were collected in the Concomitant Medication eCRF, which was completed 2-week post-randomisation or 2-week post-surgery depending on the randomised allocation and at 6- and 12-month post-randomisation. Similar to the discharge medication, the name, dose, frequency and duration of these medications were collected, and unit costs were obtained from routine sources.^12^

The unit costs used in the economic analysis are presented in online supplemental table S2, and all costs are in 2020 GBP.

The total cost for each participant was estimated by multiplying the frequency each resource was used by the participant by the corresponding unit costs. These costs were then estimated for each randomised group by dividing total participant costs in each arm by the number of participants in each arm with cost data. These costs are summarised as the average total surgery cost, average total nasal spray cost, average total medication cost, average total HUQ costs at 6 and 12 months and the overall average totalhealthcare resource use.

### Estimation of benefits

Utility scores were estimated by mapping responses to the Short Form-36 (SF-36) (collected at baseline, 6- and 12-month post-randomisation) onto the SF-6D preference-based utility index.^14^ Using the area under the curve method, these utility values were then combined with time weights to estimate QALYs for each participant.^15^ One QALY is equivalent to 1 year in the best possible health or ‘perfect’ health.

### Missing data

It was assumed that there would be some missing data given that both the HUQ and SF-36 were self-completed by participants. It was also assumed that data were missing at random, but this assumption was validated by testing whether there were any statistically significant differences in baseline characteristics between participants with missing and complete data. Chained multiple imputation methods were used in the base case analysis, which allowed simultaneous predictions for missing cost and utility data.^16^ A complete case analysis, which only included data from participants with complete HUQ and SF-36, was undertaken as a sensitivity analysis.

### Estimation of cost-effectiveness

Similar to the primary outcome, the economic analysis compared costs and outcomes for each randomised arm using data from the intention-to-treat (ITT) population.^5^ A sensitivity analysis compared costs and outcomes between the two treatments at 12 months. It is important to note that crossovers from medical management to septoplasty were permitted after the primary outcome 6-month timepoint; hence, this analysis disproportionately reduced the number of medical management participants.

Unadjusted analyses were used to estimate the average total costs and average total QALYs for each randomised arm. Adjusted analyses, using seemingly unrelated regression (SUR) and controlling for potential differences in age, gender, ethnicity, baseline SNOT-22 and baseline utility scores, were used to estimate incremental differences.^17^

Decisions on potential cost-effectiveness can be made based on the results of the incremental analysis. If one of the interventions was less costly and more effective than its comparator, it would be considered dominant and hence the preferred management strategy. However, if an intervention was not dominant, then the incremental cost per additional QALY gained was estimated; this is done by dividing incremental costs by incremental QALYs.^18^

### Sensitivity analysis

The assumptions and estimates used in the economic analysis were subject to uncertainty; hence, stochastic sensitivity analyses using non-parametric bootstrapping were used. The bootstrapping technique using a SUR model allowed us to randomly select, with replacement, costs and effects simultaneously from individuals in each of the randomised groups. Differences between these pairs were then plotted onto the cost-effectiveness plane;^19^ this process was replicated for 1000 iterations. The results of the bootstrapping process were also combined with a variety of values for an additional QALY (£0 to £50 k) to estimate the probability of each management strategy being considered cost-effective; these results were presented as cost-effectiveness acceptability curves (CEACs).^8^

### Economic model

**Figure 1:**
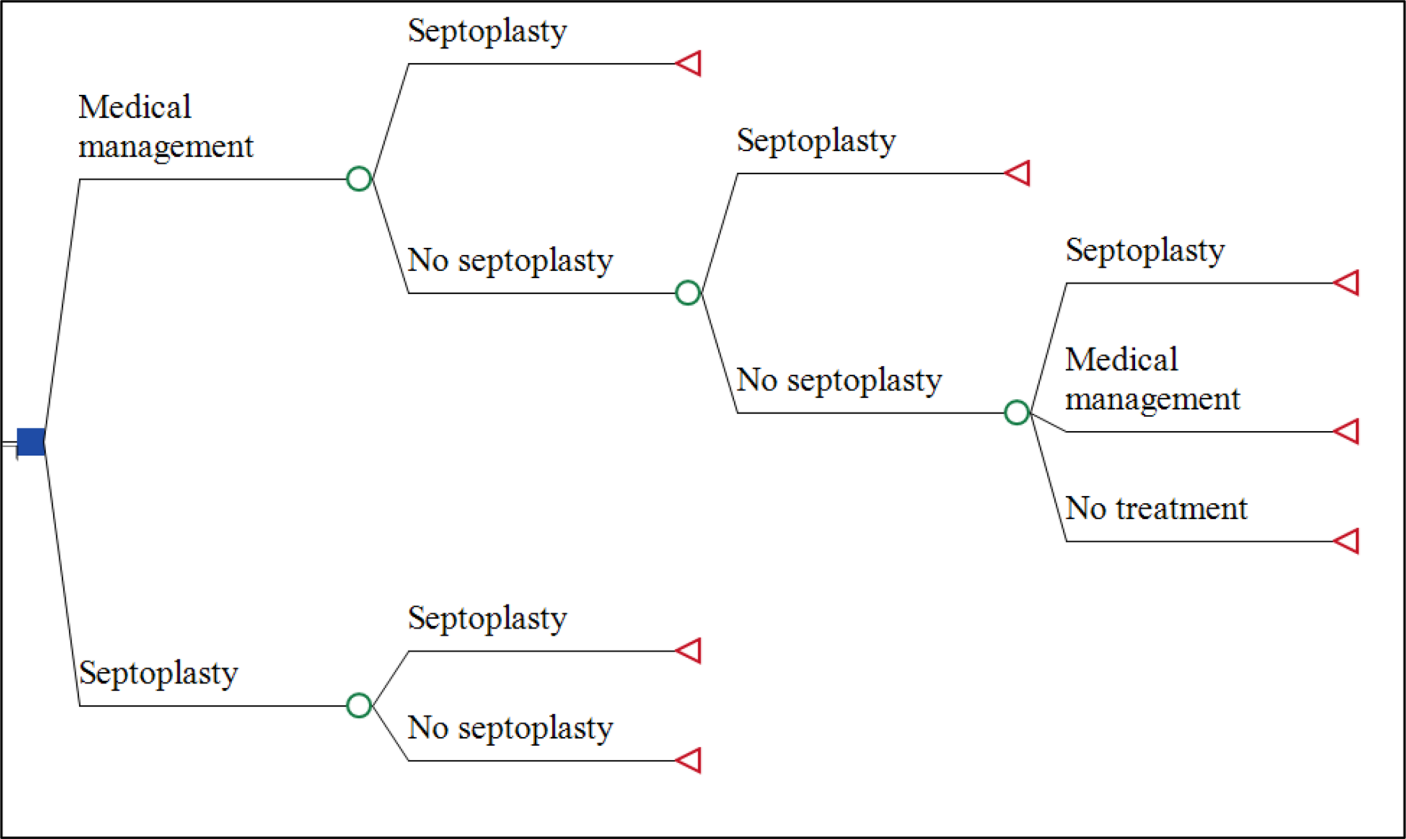
This figure is an illustration of the model structure used to estimate cost-effectiveness at 24 months.

While the results of the within-trial analysis are important to inform decisions on the short-term (12 month) cost-effectiveness of septoplasty, we anticipated that the surgical arm could be more costly and potentially more effective, but the time horizon of the trial would not be sufficient to capture these potential longer-term benefits. The trial findings, using data from the ITT population, were therefore extrapolated using a simple decision tree model in TreeAge^20^ for an additional 12 months to estimate the cost-effectiveness of immediate septoplasty compared with 6 months of medical management and the option for deferred septoplasty at 24-month post-randomisation. Figure 1 illustrates the model pathway which replicated the study.

### Costs

Intervention costs (surgery and/or nasal sprays) were assumed to be the same as those used in the base case within-trial analysis and were assigned depending on the pathway. After 12 months, it was assumed that there would be a demand for further treatments by those randomised to 6 months of medical management with the option for deferred septoplasty.

It was assumed that both randomised arms had equivalent healthcare costs at the start of the model; hence, costs incurred by the 6 months of medical management with the option for the deferred septoplasty arm were assumed between baseline and 6 months. Adjustments were made for healthcare utilisation costs for those who had septoplasty after 6 months using an OLS regression, with the same covariates as the SUR, of the reported healthcare utilisation costs between 6 and 12 months. It was assumed that healthcare utilisation costs incurred after 12 months, assigned at 6-month intervals, were equivalent to those reported during the last 6 months of the trial (ie, 12-month costs). Further adjustments to healthcare utilisation costs associated with surgery were assigned based on when during the 24-month surgery was performed.

### Utilities

The same approach used to estimate costs in the model was also adopted for utilities; the 6 months of medical management with the option for deferred septoplasty arm utility data were assumed to be the base, and adjustments, using OLS regressions, were made if surgery was performed. Utilities incurred after 12 months were assumed to be the same as those incurred in the last 6 months of the trial, and they were assigned in 6-month intervals.

### Transition probabilities

Transition probabilities were based on the probability of septoplasty for each randomised arm. Based on the trial findings, as expected, a higher proportion of those randomised to the immediate septoplasty arm underwent surgery than those in the 6 months of medical management with the option for the deferred septoplasty arm (97% vs 30%). It was assumed in the base case analysis that no additional surgeries were undertaken for those randomised to immediate septoplasty. It was assumed, based on clinical advice, that there would be a steady decline in the uptake rate of septoplasty for those in the 6 months of medical management with the option for the deferred septoplasty arm after 12 months (15%). It was also assumed, based on clinical advice, that a proportion (50%) of those randomised to 6 months of medical management with the option for deferred septoplasty who had not received septoplasty would recommence using nasal sprays to manage their deviated septum. These assumptions were explored in sensitivity analyses which varied the transition probabilities±50%. All model parameters are presented in online supplemental table S3.

### Model validation

Internal validations were undertaken by checking the model structure, calculations and data parameters, with further validation running the model for 12 months to replicate the within-trial analysis.

### Sensitivity analysis

Probabilistic sensitivity analysis (PSA) was undertaken to quantify any potential uncertainty in the model parameters, as each model parameter, except the intervention costs (surgery and nasal spray) and transition probabilities for surgery, which were fixed, had a measure of uncertainty surrounding it (SD) and was assigned a statistical distribution. The PSA facilitates the estimation of costs and effects using a set of parameters drawn from the statistical distribution using Monte Carlo simulations. Similar to the bootstrapping of the within-trial results, 1000 Monte Carlo simulations were drawn to estimate the probability of immediate septoplasty being considered cost-effective at a range of thresholds for an additional QALY.

### Patient and public involvement

Patient representatives were involved in both the design and conduct of the study, including the choice of the primary outcome and opportunity for those in medical management to discuss undergoing septoplasty after 6 months. Specifically for the economic analysis, patient representatives were consulted on and asked to review the data collection tools, particularly the HUQ, for relevance and acceptability.

## Results

Data on 307 participants (6 months of medical management with the option for delayed septoplasty (n=155), immediate septoplasty (n=152)) from the ITT population were used in the economic analysis. As expected, there was a slight decline in the response rate to both the HUQ and SF-36 over the 12-month follow-up period; however, two-thirds of participants had complete data (HUQ=66%; SF-36=68%). Missing responses to these questionnaires ranged from 2% at baseline to 30% at 12 months. Response rates to each individual question at each time point are presented elsewhere.^21^

The assumption that missing data were missing at random was validated, as there was no evidence of a difference in baseline characteristics between participants (mean difference, p-value: age −3.15, p=0.0787; gender −0.04, p value=0.5329; baseline utility −0.010, p value=0.5768)).

### Resource use and costs

The average total healthcare costs by randomised arm are presented in table 1. On average, higher costs were incurred by the immediate septoplasty arm compared with 6 months of medical management with the option for the deferred septoplasty arm (£2162 vs £973). The differences in costs were largely driven by more participants undergoing surgery in the immediate septoplasty arm (n=148) compared with the 6 months of medical management with the option for the deferred septoplasty arm (n=47). Both randomised arms reported similar healthcare resource use over the 12-month follow-up period in table 1.

**Table 1:** Average total costs (£) and effects per randomised arm

	6 months of medical management with the option for delayed septoplasty (n=155)		Immediate septoplasty (n=152)	
	N	Mean (SD)	n	Mean (SD)
Costs				
Surgery costs*	155†	593 (902)	152†	1905 (314)
Discharge medications	9	8 (7)	123	8 (7)
Nasal spray costs	155	91 (0)	152	0 (0)
HUQ costs @ 6 months	142	134 (238)	140	156 (157)
HUQ costs @ 12 months	115	143 (168)	99	97 (128)
Total HUQ costs	109	261 (292)	95	276 (234)
Medication costs	63	8 (15)	84	16 (32)
Total costs	109	930 (980)	95	2207 (358)
Total costs – multiple imputation	155	973 (1028)	152	2162 (375)
Effects				
SF-6D @ baseline	152	0.712 (0.14)	149	0.715 (0.14)
SF-6D @ 6 months	140	0.729 (0.14)	140	0.789 (0.14)
SF-6D @ 12 months	117	0.742 (0.16)	103	0.777 (0.14)
QALYs	111	0.741 (0.13)	99	0.761 (0.13)
QALYs – multiple imputation	152	0.727 (0.12)	149	0.766 (0.12)

n=total number of participants providing data.

N=total number of participants in each arm.

* Surgery costs were estimated using NHS tariff^10^.

† Number of participants who underwent surgery in each arm (septoplasty n=148; 6 months of medical management with the option for deferred surgery n=47).

HUQ, healthcare utilisation questionnaire; QALYs, quality-adjusted life years; SF-6D, Short-Form 6 Dimensions.

The potential impact of the recruitment site on the cost-effectiveness outcomes was confirmed and had no effect on costs (p=0.083) or QALYs (p=0.714).

### Effectiveness outcomes

Table 1 presents the average utility values at baseline, 6- and 12-month post-randomisation and the average total QALYs per randomised arm. On average, participants randomised to immediate septoplasty reported higher utilities and QALYs than those randomised to 6 months of medical management with the option for deferred septoplasty.

### Incremental cost-effectiveness

Table 2 presents the incremental cost-effectiveness results. On average, immediate septoplasty was more costly and more effective than 6 months of medical management with the option for deferred septoplasty. The incremental cost per QALY gained was £27 000.

**Table 2:** Cost-effectiveness of immediate septoplasty compared with 6 months of medical management with the option for deferred septoplasty at 12 months

Strategy	Cost (£) (SD) ^*^	Incremental cost (£) (95% CI) ^†^	QALYs (SD)*	Incremental QALYs (95% CI) ^†^	ICER
6 months of medical management with the option for deferred septoplasty‡	973 (810 to 1137)		0.728 (0.71 to 0.75)		
Immediate septoplasty	2162 (2102 to 2222)	1193 (1018 to 1368)	0.767 (0.75 to 0.79)	0.044 (0.03 to 0.06)	27 114

* Point estimates are based on the unadjusted analysis (costs n=307, QALYs n=301).

† Incremental results based on adjusted analysis (n=299).

‡ 30% had surgery.

ICER, incremental cost-effectiveness ratio; QALYs, quality-adjusted life years.

The results of the stochastic sensitivity analysis are presented in online supplemental figures S1 and figure 1. In online supplemental figure S1, all of the bootstrapped iterations indicate that immediate septoplasty is more costly but more effective than 6 months of medical management with the option for deferred septoplasty. The majority of the iterations are above the £20 000 threshold for an additional QALY.

**Figure 2:**
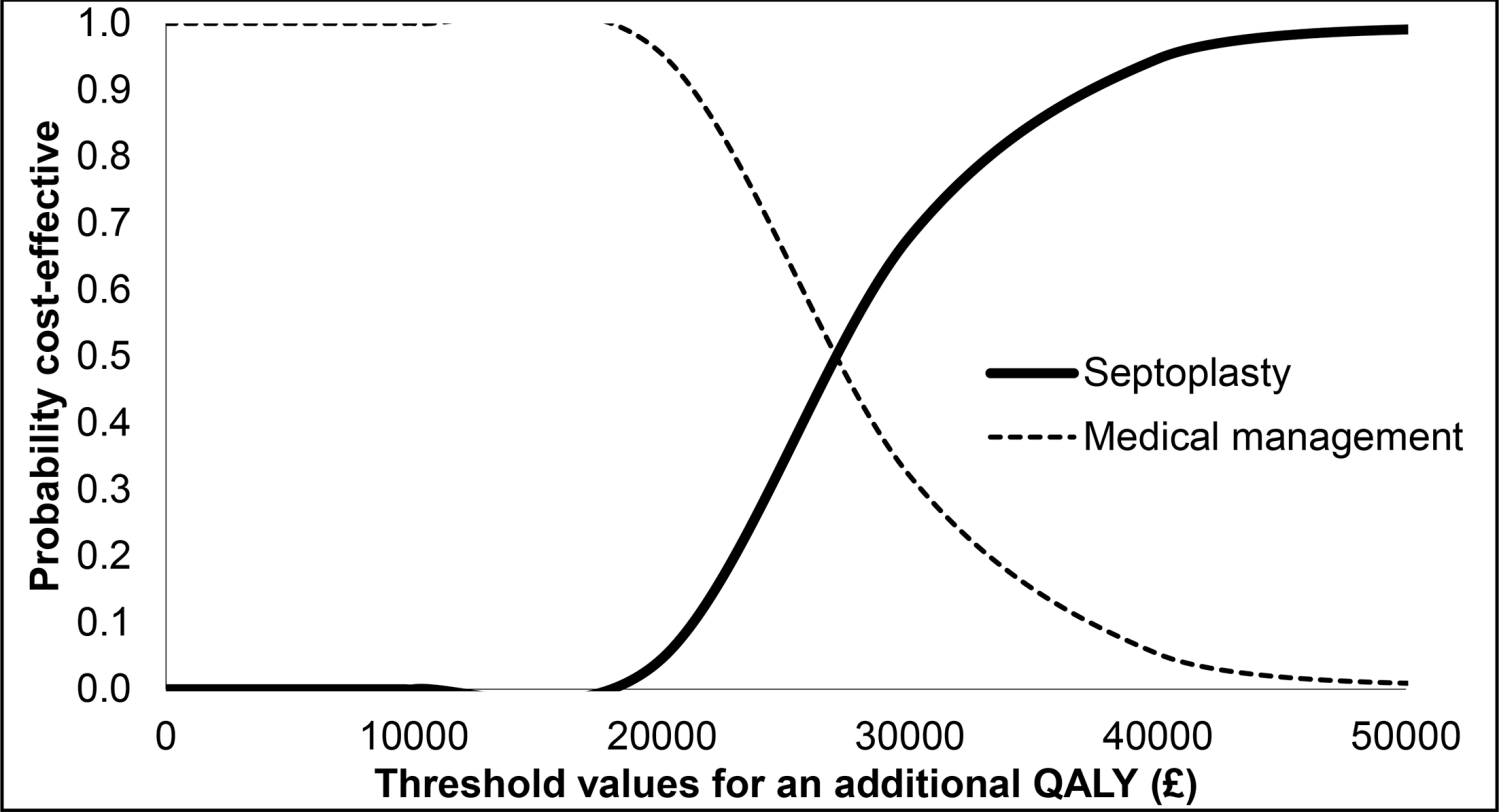
This figure shows the probability of septoplasty being considered cost-effective at different willingness-to-pay values for an additional QALY at 12 months. QALY, quality-adjusted life year.

Figure 2 illustrates the probability of immediate septoplasty being considered cost-effective over a range of willingness-to-pay thresholds for an additional QALY. The probability of immediate septoplasty being considered cost-effective increases from 0% to 99% over the willingness-to-pay thresholds considered. At a £20 000 threshold for an additional QALY, the probability of immediate septoplasty being considered cost-effective was 15%, and this increased to 68% at a £30 000 threshold.

Sensitivity analyses are presented in online supplemental table S4 (complete case analysis) and online supplemental table S5 (analysis including participants who received the treatment they were randomised to). The conclusions remain the same in that immediate septoplasty is, on average, more costly and more effective than 6 months of medical management with deferred surgical treatment at 12 months. For both analyses, the probability of immediate septoplasty being considered cost-effective increases as the threshold for an additional QALY increases. However, immediate septoplasty is unlikely to be considered cost-effective at 12 months at current thresholds for an additional QALY.

### Micro-costing surgery costs

When micro-costing was used to estimate the surgery costs instead of the tariff, the average total cost of both randomised arms reduced (6 months of medical management with the option for deferred septoplasty: £797 (95% CI £650 to £884); and immediate septoplasty was £1500 (95% CI £1453 to £1547)). The conclusions remained the same in that, on average, immediate septoplasty was more costly and more effective than 6 months of medical management with the option of deferred septoplasty at 12 months with an incremental cost per QALY of £16 682. The probability of immediate septoplasty being considered cost-effective increased to 79% at a £20 000 threshold for an additional QALY.

### Economic model

Similar to the within-trial results, at 24 months immediate septoplasty was both more costly (£833) and more effective (0.06 QALYs) compared with 6 months of medical management with the option for delayed septoplasty. The extrapolation of costs and QALYs over a longer period resulted in the reduction of the incremental cost per additional QALY to £13 221. The results of the model are presented in table 3.

**Table 3:** Cost-effectiveness of immediate septoplasty compared with 6 months of medical management with the options for deferred septoplasty at 24 months

Strategy	Cost (£)	Incremental cost (£)	QALYs	Incremental QALYs	ICER
6 months of medical management with the option for deferred septoplasty	1483		1.46		
Immediate septoplasty*	2316	833	1.53	0.06	13 221

* Assumption: 30% had surgery in the first 12 months (based on the trial results). A further 15% had surgery between 12 and 24 months (based on clinical advice).

ICER, incremental cost-effectiveness ratio; QALYs, quality-adjusted life years.

The results of the PSA estimated that immediate septoplasty had a 99% probability of being considered cost-effective at a £20 000 threshold for an additional QALY at 24 months (online supplemental figure S2).

Conclusions did not change when additional sensitivity analyses exploring the assumptions of the model were made; immediate septoplasty had a higher probability compared with 6 months of medical management with the option for deferred septoplasty of being considered cost-effective at a £20 000 threshold for an additional QALY at 24 months.

## Discussion

On average, those randomised to the immediate septoplasty arm reported higher total average healthcare costs than those randomised to the 6 months of medical management with the option for the deferred septoplasty arm. This difference in costs is largely driven by the difference in the costs of the interventions (surgery and nasal sprays), as both randomised arms reported similar primary and secondary healthcare costs over the 12-month follow-up.

At baseline, both randomised arms reported similar average utility values (0.71) based on responses to the SF-36. At 6 and 12 months, those randomised to immediate septoplasty reported higher average utility values and higher QALYs compared with those reported by 6 months of medical management with the option for deferred septoplasty. The SF-36 results are similar to the results of the other effectiveness measures collected as part of the trial,^5^ such as SNOT-22 and Nasal Obstruction and Symptom Evaluation scores, which found immediate septoplasty to be the superior treatment.

As immediate septoplasty was on average more costly and more effective compared with 6 months of medical management with the option for deferred septoplasty, an ICER was estimated. In the within-trial analysis, the ICER was £27 114 per QALY gained, and immediate septoplasty had a 15% probability of being considered cost-effective at a £20 000 threshold for an additional QALY at 12 months.

When the cost of surgery was estimated using micro-costing, conclusions remained the same in that immediate septoplasty was more costly but more effective than 6 months of medical management with the option for deferred septoplasty. However, the ICER reduced to £16 682, and the probability of immediate septoplasty being considered cost-effective at a £20 000 threshold for an additional QALY was 79% at a £20 000 threshold for an additional QALY. This result suggests that it is the cost of surgery determining cost-effectiveness at 12 months. However, these results need to be interpreted with caution.

The primary clinical outcome of the NAIROS trial was estimated at 6 months; however, it was agreed that this time horizon would be too short for the economic analysis. Hence, cost and utility data were collected for 12 months. Despite the longer follow-up for the economic data, it was still hypothesised that if immediate septoplasty was more effective than 6 months of medical management with the option for deferred septoplasty, 12 months would be insufficient for the potential benefits associated with surgery to offset the cost of surgery. A simple decision tree was used to extrapolate the trial findings beyond the 12-month follow-up period. It was agreed that a 24-month time horizon should be adopted to facilitate comparisons between our findings and other research.^22^ The model used data directly from the trial, and assumptions on further treatment for those in the 6 months of medical management with the option for deferred septoplasty arm were made based on clinical input. At 24 months, the conclusion remained the same as the within-trial analysis: immediate septoplasty was, on average, more costly and more effective than 6 months of medical management with the option for deferred septoplasty. However, the incremental cost per QALY gained was lower (£13 221), and the probability of immediate septoplasty being considered cost-effective at a £20 000 threshold for an additional QALY increased to 99% at 24 months.

The study clinics recruited a diverse range of ethnicities and ages, as well as from both sexes, reflecting septoplasty operation provision in the NHS. The wide geographic spread of recruitment from both teaching hospitals and district general hospitals as well as a range of different clinician-graded surgeons performing the trial septoplasty ensured the trial participants were representative of the general UK population.

To our knowledge, this is the second economic evaluation investigating the cost-effectiveness of septoplasty and the first to evaluate the cost-effectiveness of immediate septoplasty compared with 6 months of medical management (nasal sprays) with the option for deferred septoplasty in the management of a deviated septum.^22^ Egmond *et al* (2020) estimated the cost-effectiveness of septoplasty compared with non-surgical treatment, which consisted of non-standardised conservative management (watchful waiting and local corticosteroids).^22^ Similar to Egmond *et al* (2020), septoplasty is unlikely to be considered cost-effective at 12 months compared with medical management (Egmond *et al* (2020), 7%, versus 15% in NAIROS, assuming a £20 000 threshold for an additional QALY).^22^ Both studies concluded that 12-months it too short a timehorizon for the costs and benefits associated with septoplasty to be considered and undertook analyses at 24-months. At 24 months, both studies found that differences in costs between septoplasty and medical management decrease over time due to those in the medical management group needing further treatment, while the differences in health-related quality of life (HRQoL) between septoplasty and medical management remained or increased.^22^ Both studies concluded that septoplasty had a higher probability of being considered cost-effective at 24 months (Egmond *et al* (2020) 54% vs 99% in NAIROS, assuming a £20 000 threshold for an additional QALY).^22^

While the conclusions between both economic evaluations are consistent, caution needs to be taken when directly comparing the two studies. First, the unit costs used in the costing analysis have been gathered from both countries, with Egmond *et al* (2020) converting their costs to GBP (£) 2017. Second, both studies used different HRQoL tools to estimate QALYs. The EQ-5D-3L, which was used by Egmond *et al* (2020) has been succeeded by the EQ-5D-5L, which arguably is more sensitive to pick up potentially small but important changes in QoL. Finally, one of the strengths of the NAIROS economic evaluation was the statistical methods used in the estimation of costs and QALYs. SUR was used to estimate the difference in costs and QALYs, and this analysis is arguably more reliable for estimating the ICER with chained multiple imputation methods used to account for missing data.^16 17^

The inclusion of the economic analysis as part of the RCT supports the validity of these results, as the analysis was pre-planned, and the data used were collected directly from participants. An additional strength of this analysis is that two-thirds of participants had complete data, and statistical methods of imputation were used to maximise the data available. Reassuringly, all sensitivity analyses found similar conclusions to the primary economic analysis. However, caution needs to be taken when interpreting these results, as the randomised treatment analysis disproportionately reduced the number of participants in the 6 months of medical management with the option for the deferred septoplasty arm (n=47) compared with the immediate septoplasty arm (n=4). Also, participants randomised to 6 months of medical management were permitted to undergo septoplasty after the 6-month primary outcome timepoint. The primary economic analysis presented here used an ITT population, which is recommended for economic analyses to avoid these potential biases, as it is reflective of real-world effectiveness.^8^

There are limitations associated with the economic analysis. First, the micro-costing estimates potentially underestimated the cost of surgery, as additional costs such as overheads were not included, and these costs were only sourced from one site. Additionally, data used to inform the micro-costing exercise were only collected for participants randomised to immediate septoplasty, but 30% (n=47) of participants randomised to 6 months of medical management with the option for deferred septoplasty underwent septoplasty. This has meant that variations in surgical costs were not captured in the bootstrapping analysis, as every participant who underwent surgery was assigned the same cost. While pragmatically the trial had a 12-month follow-up, it was arguably too short a time horizon for costs and benefits associated with septoplasty to be considered. The economic model overcame this limitation; however, it should be noted that, while validity checks and sensitivity analyses were undertaken to support the validity of the longer-term economic results, data to populate the model were taken from a single study and clinical advice. It was also assumed, given the short time horizon of 24 months, that the only costs associated with the treatment failure of septoplasty were the adjustment of healthcare utilisation costs. While this is arguably a conservative assumption in favour of the immediate septoplasty arm, it was supported by the trial findings, as no participants required repeat surgery, and the assumption was made for all participants who underwent the surgery, regardless of randomised allocation.

Recommendations for further research in this area would be to consider collecting longer-term data for those who undergo septoplasty. However, despite the conservative assumptions in the economic model, there is strong evidence to support that immediate septoplasty is likely to be considered cost-effective compared with 6 months of medical management with the option for deferred septoplasty in the management of deviated nasal septum at 24 months.

## Conclusion

In the results of the within-trial analysis, immediate septoplasty is more costly and more effective than 6 months of medical management with the option for deferred septoplasty. However, at 12 months, there is uncertainty over which management strategy is preferable depending on how surgery costs were estimated. At 24 months, immediate septoplasty would be considered cost-effective in the management of a deviated nasal septum.

### Take-home message

Immediate septoplasty is more costly but more effective, in terms of QALYs gained, than 6 months of medical management with the option for deferred septoplasty in the management of deviated nasal septum. At 12 months, there is uncertainty in the probability of immediate septoplasty being considered cost-effective at a £20 000 threshold for an additional QALY, as it ranged from 15% to 79%. However, when a 24-month time horizon was adopted, immediate septoplasty is the preferred management strategy with a 99% probability of being considered cost-effective at a £20 000 threshold for an additional QALY.

## Supplementary material

10.1136/bmjopen-2025-107402 online supplemental file 1

10.1136/bmjopen-2025-107402 online supplemental file 2

10.1136/bmjopen-2025-107402 online supplemental file 3

10.1136/bmjopen-2025-107402 online supplemental file 4

10.1136/bmjopen-2025-107402 online supplemental file 5

10.1136/bmjopen-2025-107402 online supplemental file 6

10.1136/bmjopen-2025-107402 online supplemental file 7

## Data Availability

Data are available upon reasonable request.
