# Supplementary material for: Cost-effectiveness of immediate septoplasty versus medical management with the option for delayed septoplasty for nasal airways obstruction: a multicentre, open-label, randomised controlled trial
Source: BMJ Open. 2026 Jul 6;16(7):e107402. doi: 10.1136/bmjopen-2025-107402 (PMC13343045; doi:10.1136/bmjopen-2025-107402)
Supplement: online supplemental file 1 [file bmjopen-16-7-s001.docx]

**Figure S1 Cost-effectiveness plane for septoplasty versus medical management at 12-months**
