# Supplementary material for: Cost-effectiveness of immediate septoplasty versus medical management with the option for delayed septoplasty for nasal airways obstruction: a multicentre, open-label, randomised controlled trial
Source: BMJ Open. 2026 Jul 6;16(7):e107402. doi: 10.1136/bmjopen-2025-107402 (PMC13343045; doi:10.1136/bmjopen-2025-107402)
Supplement: online supplemental file 2 [file bmjopen-16-7-s002.docx]

**
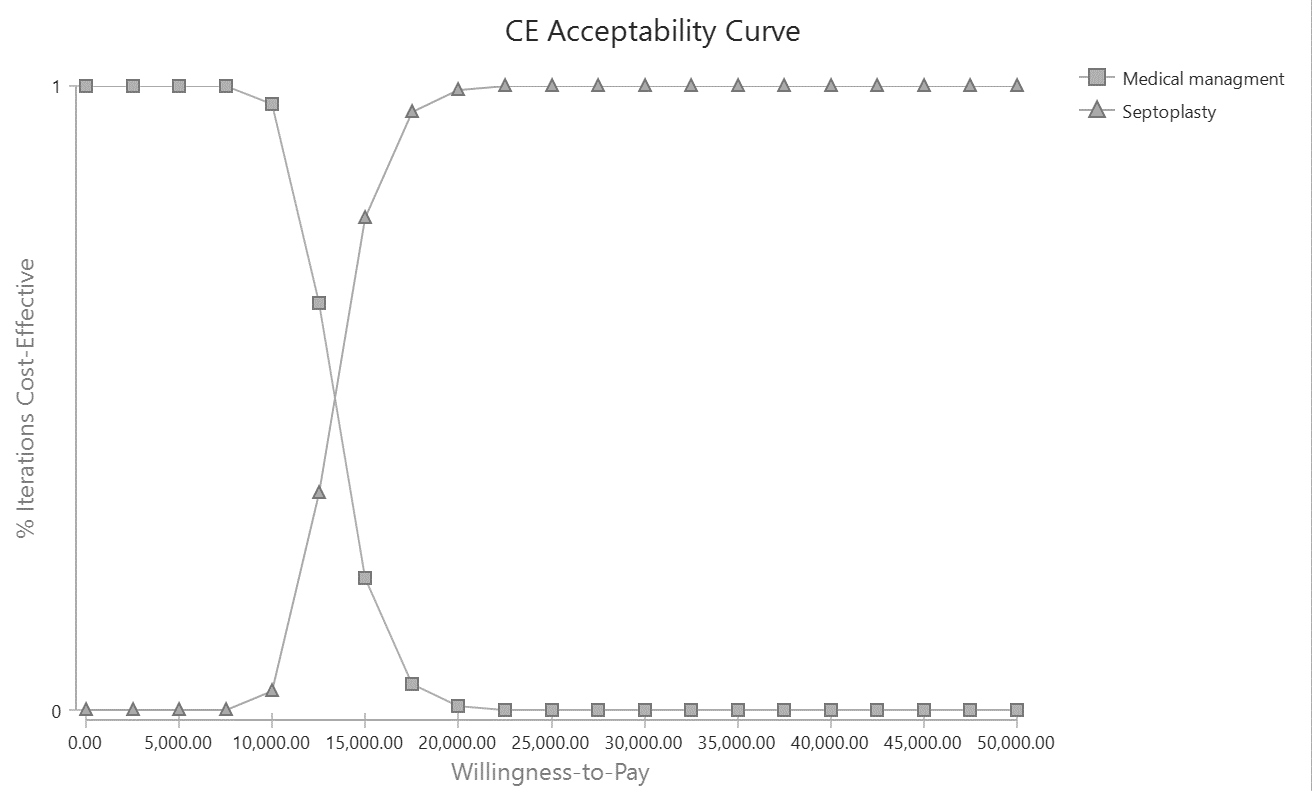
**

**Figure S2 Cost-effectiveness acceptability curve for septoplasty versus medical management at 24-months**
