# Supplementary material for: Cost-effectiveness of immediate septoplasty versus medical management with the option for delayed septoplasty for nasal airways obstruction: a multicentre, open-label, randomised controlled trial
Source: BMJ Open. 2026 Jul 6;16(7):e107402. doi: 10.1136/bmjopen-2025-107402 (PMC13343045; doi:10.1136/bmjopen-2025-107402)
Supplement: online supplemental file 3 [file bmjopen-16-7-s003.docx]

Table S1 Micro-costing unit costs

| **Resource use** | **Unit cost (£)** | **Source** |
| --- | --- | --- |
| Theatre cost (per min) | 13.75 |  |
| Ward cost (total recovery time) | 60.00 |  |
| Pre-assessment cost | 9.50 |  |
| Consultant (per min) | 1.90 |  |
| Registrar (per min) | 0.83 |  |
| Scrub nurse (per min) | 0.83 |  |
| ODP (per min) | 0.67 |  |
| Healthcare assistant (per min) | 0.23 |  |
| Anaesthetist assistant (per min) | 0.67 |  |
| Septoplasty tray (cost per use) | 5.37 | Assumed 10year lifespan, 3.5% discount for equivalent annual cost and used on average once/week |
| Septoplasty tray (autoclave) | 40.00 |  |
| Anaesthetic consumables | 46.15 |  |
| Surgical consumable | 7.14 |  |
| Gowns – TSSU | 2.00 |  |
