# Supplementary material for: Cost-effectiveness of immediate septoplasty versus medical management with the option for delayed septoplasty for nasal airways obstruction: a multicentre, open-label, randomised controlled trial
Source: BMJ Open. 2026 Jul 6;16(7):e107402. doi: 10.1136/bmjopen-2025-107402 (PMC13343045; doi:10.1136/bmjopen-2025-107402)
Supplement: online supplemental file 4 [file bmjopen-16-7-s004.docx]

Table S2 Unit costs

| **Resource use** | **Unit cost** | **Source** | **Notes** |
| --- | --- | --- | --- |
| GP consultation | £34.00 | PSSRU 2020 |  |
| Practice nurse consultation | £10.50 | PSSRU 2020  PSSRU 2015 | Assumed to be a 15min consultation |
| Nurse telephone consultation | £4.20 | PSSRU 2020  PSSRU 2015 | Assumed to be a 6min consultation |
| GP telephone consultation | £8.00 | PSSRU 2020 |  |
| NHS 111/NHS 24 | £15.05 | PSSRU 2020 | GP led triage |
| GP home visit | £49.02 | PSSRU 2020  PSSRU 2015 | Assumed to be an 11.4min consultation |
| Nurse home visit | £17.50 | PSSRU 2020  PSSRU 2015 | Assumed to be a 25 minute consultation |
| A&E visit | £182 | NHS reference costs 2019/2020 |  |
| Outpatient visit | £147 | NHS reference costs 2019/2020 |  |
| ENT outpatient visit | £112 | NHS reference costs 2019/2020 |  |
| Hospital admission (inpatient or day patient) | £378 | NHS reference costs 2019/2020 | Assumed to be regular day/night admission |
| Septoplasty | £1956 | NHS reference costs 2019/2020 | Daycase procedure |
