# Supplementary material for: Cost-effectiveness of immediate septoplasty versus medical management with the option for delayed septoplasty for nasal airways obstruction: a multicentre, open-label, randomised controlled trial
Source: BMJ Open. 2026 Jul 6;16(7):e107402. doi: 10.1136/bmjopen-2025-107402 (PMC13343045; doi:10.1136/bmjopen-2025-107402)
Supplement: online supplemental file 5 [file bmjopen-16-7-s005.docx]

Table S3 Model parameters

| **Model parameters** | **Mean (SD)** | **Distribution** |
| --- | --- | --- |
| **Costs** | | |
| Nasal spray | 91 (-) | No assumptions made on distribution as this was a fixed cost |
| Surgery | 1956 (-) | No assumptions made on distribution as this was a fixed cost |
| Health utilisation costs @ 6 months (multiple imputed) | 138 (235) | Gamma |
| Health utilisation costs @ 12months (multiple imputed) | 148 (156) | Gamma |
| Health utilisation costs adjustment @ 6 months for those in the septoplasty arm who had surgery | +21 (23) | Gamma |
| Health utilisation costs adjustment @ 12 months for those in the either arm who had surgery | -61 (18) | Gamma |
|  |  |  |
| **Utilities** | | |
| Baseline | 0.720 (0.14) | Beta |
| Utility @ 6 months (multiple imputed) | 0.728 (0.14) | Beta |
| Utility @ 12 months (multiple imputed) | 0.735 (0.15) | Beta |
|  |  |  |
| Utility adjustment for those who had surgery in the septoplasty arm @ 6 months who had surgery | +0.063 (0.012) | Beta |
| Utility adjustment for those in either arm who had surgery 12 months | +0.068  (0.14) | Beta |
| **Transition probabilities** | | |
| Probability of having surgery (medical management) @ 12 months | 0.30 | No assumptions made on distribution as based on trial data |
| Probability of having surgery (medical management arm only) @ 24 months | 0.15 | No assumptions made on distribution as based on clinical advice but explored in sensitivity analyses |
| Probability of having surgery (medical management arm only) @ 36 months | 0.075 | No assumptions made on distribution as based on clinical advice but explored in sensitivity analyses |
| Probability of having medical management at 24 and 36 months (medical management arm only) | 0.50 | No assumptions made on distribution as based on clinical advice but explored in sensitivity analyses |
| Probability of having surgery (septoplasty arm only) @ 12 months | 0.97 | No assumptions made on distribution as based on trial data |
