# Supplementary material for: Cost-effectiveness of immediate septoplasty versus medical management with the option for delayed septoplasty for nasal airways obstruction: a multicentre, open-label, randomised controlled trial
Source: BMJ Open. 2026 Jul 6;16(7):e107402. doi: 10.1136/bmjopen-2025-107402 (PMC13343045; doi:10.1136/bmjopen-2025-107402)
Supplement: online supplemental file 6 [file bmjopen-16-7-s006.docx]

**Table S4** Cost-effectiveness of septoplasty compared with medical management at 12-months using complete case dataset

| **Strategy** | **Cost (£) (SD) ^a^** | **Incremental cost (£) (95% CI) ^b^** | **QALYs (SD) ^a^** | **Incremental QALYs**  **(95% CI) ^b^** | **ICER** | **Probability of being cost-effective** | | | | |
| --- | --- | --- | --- | --- | --- | --- | --- | --- | --- | --- |
|  |  |  |  |  |  | **£0** | **£10k** | **£20k** | **£30k** | **£50k** |
| Medical management | 930  (744 to 1116) |  | 0.741  (0.72 to 0.76) |  |  | 1.00 | 1.00 | 1.00 | 0.77 | 0.17 |
| Septoplasty | 2207  (2134 to 2280 | 1308  (1100 to 1515) | 0.761  (0.74 to 0.79) | 0.035  (0.02 to 0.05) | 37,371 | 0.00 | 0.00 | 0.00 | 0.23 | 0.83 |

^a^ point estimates are based on the unadjusted analysis (costs n=204, QALYs n=210; ^b^ Incremental results based on adjusted analysis (n=199)*;* CI = confidence interval; ICER = incremental cost-effectiveness ratio; QALY = quality-adjusted life year; SD = standard deviation.
