# Supplementary material for: Cost-effectiveness of immediate septoplasty versus medical management with the option for delayed septoplasty for nasal airways obstruction: a multicentre, open-label, randomised controlled trial
Source: BMJ Open. 2026 Jul 6;16(7):e107402. doi: 10.1136/bmjopen-2025-107402 (PMC13343045; doi:10.1136/bmjopen-2025-107402)
Supplement: online supplemental file 7 [file bmjopen-16-7-s007.docx]

**Table S5** Cost-effectiveness of septoplasty compared with medical management at 12-months based on treatment received

| **Strategy** | **Cost (£) (SD) ^a^** | **Incremental cost (£) (95% CI) ^b^** | **QALYs (SD) ^a^** | **Incremental QALYs**  **(95% CI) ^b^** | **ICER** | **Probability of being cost-effective** | | | | |
| --- | --- | --- | --- | --- | --- | --- | --- | --- | --- | --- |
|  |  |  |  |  |  | **£0** | **£10k** | **£20k** | **£30k** | **£50k** |
| Medical management | 333 (28) |  | 0.728 (0.01) |  |  | 100% | 100% | 100% | 96% | 19% |
| Septoplasty | 2213 (18) | 1878  (1819 to 1938) | 0.769 (0.01) | 0.045  (0.03 to 0.06) | 41,733 | 0% | 0% | 0% | 4% | 81% |

^a^ point estimates are based on the unadjusted analysis (costs n=256, QALYs n=251; ^b^ Incremental results based on adjusted analysis (n=249)*;* CI = confidence interval; ICER = incremental cost-effectiveness ratio; QALY = quality-adjusted life year; SD = standard deviation.
