# Supplementary material for: Cost-effectiveness of immediate septoplasty versus medical management with the option for delayed septoplasty for nasal airways obstruction: a multicentre, open-label, randomised controlled trial
Source: BMJ Open. 2026 Jul 6;16(7):e107402. doi: 10.1136/bmjopen-2025-107402 (PMC13343045; doi:10.1136/bmjopen-2025-107402)
Supplement: Supplementary file 8 [file bmjopen-2025-107402.reviewer_comments.pdf]

PEER REVIEW HISTORY

BMJ Open publishes all reviews undertaken for accepted manuscripts. Reviewers are asked to complete a checklist review form and are provided with free text boxes to elaborate on their assessment. These free text comments are reproduced below.

ARTICLE DETAILS

Title (Provisional)

Cost-effectiveness of immediate septoplasty versus medical management with the option for deferred septoplasty for nasal airways obstruction: a multicentre, open label, randomised controlled trial

Authors

Homer, Tara Marie; Carrie, Sean; Fouweather, Tony; Teare, Dawn; Waugh, Nichola; O'Hara, James; Wilson, Janet A; Ternent, Laura

---

VERSION 1 - REVIEW

**Reviewer**	**1**
**Name**	**Youssef, Ahmed**
**Affiliation**	**Alexandria Medical School, Otolaryngology/Head and Neck Surgery**
**Date**	**27-Aug-2025**
**COI**	**None**

It is an interesting study discussing the cost and effectiveness of septoplasty versus medical treatment on nasal obstruction in patients with deviated nasal septum, I agree with cost and long term effectiveness of surgery, however in addition to that short time frame for assessment(12 Months), I would argue that septoplasty done for right patient will be even more cost effective compared to long term medical management.... in addition to potential long term local effects of these medications. Despite being simple, I really appreciate it is one of few studies addressing this topic.

**Reviewer**	**2**
**Name**	**Elden, Halil**
**Affiliation**	**Sakarya University**
**Date**	**02-Oct-2025**
**COI**	**None**

The manuscript addresses a clinically relevant and timely question regarding the cost-effectiveness of septoplasty compared to medical management. The effort to incorporate prospective data collection and a model-based analysis is commendable. However, there are substantial methodological and reporting limitations that affect the strength and generalisability of the findings.

Major concerns;

1- The study population is relatively small for an economic evaluation, limiting the reliability and external validity of the conclusions. Larger cohorts are usually required to capture sufficient variability and provide more robust cost-effectiveness estimates.

2- While QALYs and SF-36 utilities are reported, more detailed presentation of disease-specific measures such as SNOT-22 or NOSE scores would have been essential. These measures are directly relevant to patient experience, and their integration would strengthen the link between clinical effectiveness and economic outcomes.

In conclusion, the research question is important, and the study design has notable strengths. Nevertheless, due to the concerns outlined above—particularly the limited sample size and insufficient detail in clinical outcome reporting—I believe the manuscript cannot be recommended for publication in its current form.

**Reviewer**	**3**
**Name**	**Sunnergren, Ola**
**Affiliation**	**University of Gothenburg, Otorhinolaryngology**
**Date**	**04-Oct-2025**
**COI**	**None**

Thank you for a very interesting and well-written manuscript. I have a few comments and questions, which are presented in the attached PDF.

**Reviewer**	**4**
**Name**	**Kelson, Zoe**
**Affiliation**	**University of Exeter, Mathematics**
**Date**	**29-Oct-2025**
**COI**	**None**

This multicentre, open label, randomised, controlled trial aims to estimate the cost-effectiveness of septoplasty compared with medical management in individuals with nasal obstruction associated with septal deviation.

Reviewer Comments:

"Study registration: ISRCTN16168569 "

The authors have suitably registered the study.

"The economic evaluation reported adheres to the Consolidated Health Economic Evaluation Reporting Standards (CHEERS)"

Thanks for providing a copy of the CHEERS checklist.

"Further details of the trial design, inclusion criteria and clinical effectiveness outcomes, including the primary outcome which was estimated at 6 months, are presented elsewhere "

Can the authors please provide a summary in brief here?

Can a copy of the original trial protocol please be provided?

"All analyses were prespecified in a health economics analysis plan"

Can this analysis plan please be provided?

"Adjusted analysis was used to estimate incremental costs and QALYs. A model-based analysis was used to extrapolate costs and effects to 24-months." [Abstract]

Can the authors please further specify these analyses in the Abstract?

"Septoplasty had an incremental cost per QALY gained of £27,114 in the basecase analysis which decreased to £16,682 when micro-costing was used to estimate surgery costs." [Abstract]

Can confidence intervals please be reported in the Abstract?

"17 otolaryngology clinics in the UK, recruiting from January 2018 until December 2019. "

and

"The NAIROS trial was conducted across 17 otolaryngology clinics in England, Scotland and Wales. "

Can the authors please further comment on whether the trial data can be considered to be representative?

Can the authors please explore whether there are differences by clinic?

"Participants were randomised to receive either septoplasty within 12 weeks of recruitment or medical management (nasal steroid and saline spray) for six months. "

Can the authors please confirm if there were any stratification variables in the original trial randomisation?

"In the basecase analysis, the cost of surgery was assumed to be the daycase tariff for septoplasty (CA11A) reported in the National Schedule of Reference Costs (10) and this cost was assigned to all participants who received the surgical intervention. A sensitivity analysis

adopted a micro-costing technique using information collected in the electronic Case Report Form (eCRF), which was completed only for those randomised to septoplasty who received the surgical intervention"

The authors do well to undertake a sensitivity analysis on the computation of costs.

"The eCRF collected information on the duration of the surgery, the grade of the senior surgeon and senior anaesthetist present during the surgery, discharge medication, and the duration of the participants' admission. "

and

"The name, dose, frequency, and duration of all discharge medication was reported in the eCRF and the costs of these medication were collected from routine sources"

and

"A Health Utilisation Questionnaire (HUQ), designed based on previous studies and feedback from the Trial Management Group and Patient and Public Involvement (PPI) representatives, was completed by participants at baseline, 6 and 12-months post-randomisation."

and

"Unit costs of healthcare resources were obtained from routine sources "

Can the amount of missing data for each variable please be clearly reported?

"The unit costs used in the economic analysis are presented in supplementary material Table S2 and all costs are in 2020 Great British pounds (GBP). "

The authors have suitably tabulated costs, and provided a description of the assumptions, variation, and source.

"The total cost for each participant was estimated by multiplying the frequency each resource used by the participant by the corresponding unit costs. These costs were then estimated for each randomised group by dividing the total cost per participant in each arm by the number of participants in each arm with cost data. These costs are summarised in Table 1 as the average total surgery cost, average total nasal spray cost, average total medication cost, average total HUQ costs at 6 and 12 months, and the overall average total healthcare resource use"

Can the authors please consider exploring the impact of specific costs by applying one-way sensitivity analyses?

"It was assumed that there would be some missing data given that both the HUQ and SF-36 were self-completed by participants. It was also assumed that data were missing at random, but this assumption was validated by testing whether there were any statistically significant differences in baseline characteristics between participants with missing and complete data. Chained multiple imputation methods were used in the basecase analysis, which allowed simultaneous predictions for missing cost and utility data"

Can the authors please confirm if this methodology is consistent with the trial study protocol and pre-specified health economic analysis plan?

Can a sensitivity analysis on the approach taken for handling missing data please be considered?

"Similar to the primary outcome the economic analysis used data from the intention to-treat (ITT) population"

Can the authors please consider exploring a per protocol (PP) sensitivity anaysis?

"The assumptions and estimates used in the economic analysis were subject to uncertainty hence stochastic sensitivity analyses using non-parametric bootstrapping were used. The bootstrapping technique using a SUR model allowed us to randomly select, with replacement, costs and effects simultaneously from individuals in each of the randomised groups. Differences between these pairs was then plotted on to the cost-effectiveness plane (19), this process was replicated for 1000 iterations. The results of the bootstrapping process were also combined with a variety of values for an additional QALY (£0 to £50k) to estimate the probability of each management strategy being considered cost-effective, these results were presented as cost effectiveness acceptability curves (CEACs)"

and

"Probabilistic sensitivity analysis (PSA) was undertaken to quantify any potential uncertainty in the model parameters as each model parameter, except the intervention costs (surgery and nasal spray) and transition probabilities for surgery which were fixed, had a measure of uncertainty surrounding it (SD) and was assigned a statistical distribution. The PSA facilitates the estimation of costs and effects using a set of parameters drawn from the statistical distribution using Monte Carlo simulations. Similarly to the bootstrapping of the within-trial results, 1000 Monte Carlo simulations were drawn to estimate the probability of septoplasty being considered cost-effective at a range of thresholds for an additional QALY."

Valuable additional analyses exploring uncertainty have been performed.

"The trial findings were therefore extrapolated using a simple decision tree model in TreeAge (20) for an additional 12 months to estimate the cost-effectiveness of septoplasty compared with medical management at 24-months post-randomisation"

Can the outcomes of this extrapolation analysis, with uncertainty of the projection estimates quanitifed and communicated, please be shown in a Figure(s)?

"Transition probabilities were based on the probability of septoplasty for each randomised arm "

and

"These assumptions were explored in sensitivity analyses which varied the transition probabilities +/- 50%. All model parameters are presented in Table S3. "

The authors do well to explore uncertainty in transition probabilities.

"Data on 307 participants (medical management (N=155), septoplasty (N=152)) from the ITT population were used in the economic analysis"

Can the authors please communicate the basis for the original sample size calculation?

"Figure 2 illustrates the probably of septoplasty being considered cost-effective over a range of willingness to pay thresholds for an additional QALY"

Can the application of willingness to pay (WTP) please also be specified in the methods section?

"Table 3 Cost-effectiveness of septoplasty compared with medical management 13 at 24-months"

Can confidence intervals please be reported here?

Can the computation of ICER please be specified in the methods?

"Data to inform the micro-costing of septoplasty was only collected from those randomised to septoplasty hence variations in surgical costs could not be captured in the bootstrapping.
• The 12-month trial follow-up, while pragmatic, was arguably too short for the full consideration of costs and benefits associated with a surgical intervention to be considered" [Strengths and Limitations of this Study]

and

"There are limitations associated with the economic analysis. Firstly, the micro costing estimates potentially underestimated the cost of surgery as additional costs such as overheads were not included and these costs were only sourced from one site. Additionally, data used to inform the micro-costing exercise was only collected for participants randomised to septoplasty, but 30% (n=47) of participants randomised to medical management underwent septoplasty. This has meant that variations in surgical costs were not captured in the bootstrapping analysis as every participant who underwent surgery was assigned the same cost. While pragmatically the trial had a 12-month follow-up, it was arguably too short a time horizon for costs and benefits associated with septoplasty to be considered. The economic model overcame this limitation however it should be noted that while validity checks and sensitivity analyses were undertaken to support the validity of the longer-term economic results, data to populate the model was taken from a single study and clinical advice"

The authors provide a discussion on the study limitations.

VERSION 1 - AUTHOR RESPONSE

**Reviewer 1**	
It is an interesting study discussing the cost and effectiveness of septoplasty versus medical	Thank you for your feedback.

treatment on nasal obstruction in patients with deviated nasal septum, I agree with cost and long term effectiveness of surgery, however in addition to that short time frame for assessment(12 Months), I would argue that septoplasty done for right patient will be even more cost effective compared to long term medical management.... in addition to potential long term local effects of these medications. Despite being simple, I really appreciate it is one of few studies addressing this topic.	
**Reviewer 2**	
The manuscript addresses a clinically relevant and timely question regarding the cost-effectiveness of septoplasty compared to medical management. The effort to incorporate prospective data collection and a model-based analysis is commendable. However, there are substantial methodological and reporting limitations that affect the strength and generalisability of the findings.<br>Major concerns;<br>1- The study population is relatively small for an economic evaluation, limiting the reliability and external validity of the conclusions. Larger cohorts are usually required to capture sufficient variability and provide more robust cost-effectiveness estimates.	Thank you for your comments.<br><br>The population for the economic evaluation was constrained by the number of participants randomised to the study. The sample size of this study was based on 90% power to detect a minimal clinically important difference of 9 points on SNOT-22. It is common with economic evaluations conducted alongside RCTs that the sample size is based on the primary outcome of the study not the economic evaluation.<br><br>It is important to note that while larger cohorts can provide more robust results, this is not always necessary for economic evaluation which are conducted alongside RCTs. Economic evaluation does not attempt to find a statistically significant difference in costs and outcomes, it is based on the assumption of the balance of probabilities. Estimates for costs, effects, and cost-effectiveness are presented alongside measures of uncertainty which helps to inform decision-makers when information is incomplete. Instead of providing more certainty, which additional participants may (or may not) provide, economic evaluation assesses the data available to estimate the probability of one treatment option of providing more benefits compared with another (i.e., probability of being cost-effective).<br><br>Specifically, in this study, potential uncertainty in our results was extensively explored using

	deterministic, stochastic, and probabilistic sensitivity analyses hence we believe our conclusions are robust and provide strong evidence on the cost-effectiveness of septoplasty compared with medical management.
While QALYs and SF-36 utilities are reported, more detailed presentation of disease-specific measures such as SNOT-22 or NOSE scores would have been essential. These measures are directly relevant to patient experience, and their integration would strengthen the link between clinical effectiveness and economic outcomes.	While the SNOT-22 and NOSE scores were outcomes of the trial they were not outcome measures in the economic evaluation presented here. The results of the SNOT-22 and NOSE scores have been reported in the main trial paper published in the BMJ and referenced throughout this manuscript (reference #5).<br><br>However, the discussion has been updated on p.22 lines 4-6 to specifically reference the SNOT-22 and NOSE scores. "The SF-36 results are similar to the results of the other effectiveness measures collected as part of the trial (5), such as SNOT-22 and NOSE scores, which found septoplasty to be the superior treatment.
**Reviewer 3**	
Thank you for a very interesting and well-written manuscript. I have a few comments and questions, which are presented in the attached PDF.	Thank you for your feedback. Unfortunately, the comments on the PDF were not shared with us. They were requested on the 17th December 2025 and the editorial office responded on 24th December 2025 to say that reviewer 3 no longer had the file. The editorial office also advised that they shared this information with the editor, who acknowledged that we are unable to respond to any further comments from Reviewer 3.
**Reviewer 4**	
Study registration: ISRCTN16168569 "<br>The authors have suitably registered the study.	Thank you
"The economic evaluation reported adheres to the Consolidated Health Economic Evaluation Reporting Standards (CHEERS)"<br>Thanks for providing a copy of the CHEERS checklist.	You're welcome.
"Further details of the trial design, inclusion criteria and clinical effectiveness outcomes,	Please refer to reference #7 and #5.

including the primary outcome which was estimated at 6 months, are presented elsewhere " Can the authors please provide a summary in brief here?	
Can a copy of the original trial protocol please be provided?	The study protocol has been included as reference #7 and can be accessed https://pubmed.ncbi.nlm.nih.gov/32054508/
"All analyses were prespecified in a health economics analysis plan" Can this analysis plan please be provided?	Yes, the health economics plan can be shared.
"Adjusted analysis was used to estimate incremental costs and QALYs. A model-based analysis was used to extrapolate costs and effects to 24-months." [Abstract] Can the authors please further specify these analyses in the Abstract?	The abstract has been updated on p.2 lines 17 and 18. "Seemingly unrelated regression was used to estimate incremental costs and QALYs"
"Septoplasty had an incremental cost per QALY gained of £27,114 in the basecase analysis which decreased to £16,682 when micro-costing was used to estimate surgery costs." [Abstract] Can confidence intervals please be reported in the Abstract?	No, confidence intervals cannot be provided for the incremental cost per QALY gained. Uncertainty in the estimate of the incremental cost per QALY gained is presented as the probability of the septoplasty being considered cost-effective.
"17 otolaryngology clinics in the UK, recruiting from January 2018 until December 2019. " and "The NAIROS trial was conducted across 17 otolaryngology clinics in England, Scotland and Wales. " Can the authors please further comment on whether the trial data can be considered to be representative?	The study clinics recruited a diverse range of ethnicities and ages, as well as from both sexes, reflecting septoplasty operation provision in the NHS. The wide geographic spread of recruitment from both Teaching Hospitals and District General Hospitals as well as a range of different clinician graded surgeons performing the trial septoplasty ensured the trial participants were representative of the general UK population.
Can the authors please explore whether there are differences by clinic?	Similar to the primary outcome analysis (reported in Reference #5) we explored the impact of the recruitment site on the cost-effectiveness outcomes and can confirm it had no effect on costs ($p= 0.083$) or QALYs ($p= 0.714$).
"Participants were randomised to receive either septoplasty within 12 weeks of recruitment or medical management (nasal steroid and saline spray) for six months. " Can the authors please confirm if there were any stratification variables in the original trial	As reported in the main trial paper (see reference #5) randomisation was stratified by sex and baseline severity of symptoms according to scores on the NOSE scale: 30-50 (moderate), 55-75 (severe), 80-100 (extreme).

randomisation?	
"In the basecase analysis, the cost of surgery was assumed to be the daycase tariff for septoplasty (CA11A) reported in the National Schedule of Reference Costs (10) and this cost was assigned to all participants who received the surgical intervention. A sensitivity analysis adopted a micro-costing technique using information collected in the electronic Case Report Form (eCRF), which was completed only for those randomised to septoplasty who received the surgical intervention"<br>The authors do well to undertake a sensitivity analysis on the computation of costs.	Thank you. We agree that given surgery was the main cost driver, as mentioned on p.16 lines 17-20, that it was important to undertake this analysis to explore how sensitive our conclusions were to changes in the cost of surgery.
"The eCRF collected information on the duration of the surgery, the grade of the senior surgeon and senior anaesthetist present during the surgery, discharge medication, and the duration of the participants' admission. "<br>and<br>"The name, dose, frequency, and duration of all discharge medication was reported in the eCRF and the costs of these medication were collected from routine sources"<br>and<br>"A Health Utilisation Questionnaire (HUQ), designed based on previous studies and feedback from the Trial Management Group and Patient and Public Involvement (PPI) representatives, was completed by participants at baseline, 6 and 12-months post-randomisation."<br>and<br>"Unit costs of healthcare resources were obtained from routine sources "<br>Can the amount of missing data for each variable please be clearly reported?	For brevity overall response rates to questionnaires are not provided in this manuscript.<br><br>The response rate for each individual variable at each time point is available online. Please see https://www.journalslibrary.nihr.ac.uk/hta/MVFR4028<br>Appendix 2, Tables 74-76.<br><br>The text on p.16 lines 6-7 have been updated to signpost the reader. "Response rates to each individual question at each timepoint are presented elsewhere(21)."
"The unit costs used in the economic analysis are presented in supplementary material Table S2 and all costs are in 2020 Great British pounds (GBP). "<br>The authors have suitably tabulated costs, and provided a description of the assumptions, variation, and source.	Thank you.
"The total cost for each participant was estimated by multiplying the frequency each resource used by the participant by the corresponding unit costs. These costs were then estimated for each randomised group by dividing the total cost per participant in each arm by the number of participants in each arm with cost data. These costs are summarised in Table 1 as the average total surgery cost, average total nasal spray cost, average total medication cost, average total HUQ costs at 6 and 12 months, and the overall average	As presented in Table 1 and discussed above, the cost of surgery is the main driver of costs in the economic evaluation. The uncertainty in the estimation of this cost and its impact on cost-effectiveness has been explored in sensitivity analyses as reported on p.20 lines 7-14.

total healthcare resource use"<br>Can the authors please consider exploring the impact of specific costs by applying one-way sensitivity analyses?	
"It was assumed that there would be some missing data given that both the HUQ and SF-36 were self-completed by participants. It was also assumed that data were missing at random, but this assumption was validated by testing whether there were any statistically significant differences in baseline characteristics between participants with missing and complete data. Chained multiple imputation methods were used in the basecase analysis, which allowed simultaneous predictions for missing cost and utility data"<br>Can the authors please confirm if this methodology is consistent with the trial study protocol and pre-specified health economic analysis plan?	Yes, this is consistent with what was reported in the trial protocol and HEAP. Section 3.5 of the HEAP stated "When dealing with trial data there are a number of potential issues that will need to be dealt with such as missing data and not adhering to the protocol.<br>The focus of the missing data analysis will be data that is missing because the eCRF and/or participant questionnaires were not fully completed. As with all trial-based data collection, it is expected that not all participants who enter the study will complete all questionnaires and/or all of the questions in each questionnaires, therefore missing data is expected. The methods of imputation used will be determined once the full trial dataset is available, as the nature of the missing data will determine which method is most appropriate. Briggs et al. (2003) have argued that statistical imputations are accurate methods of imputation as they generate more robust standard deviations [20]. An alternative approach would be to conduct a complete case analysis, which only includes data on participants with data at all scheduled time points in the analysis."<br><br>The results of the complete case analysis have been included in the manuscript and are presented in Table S4.
Can a sensitivity analysis on the approach taken for handling missing data please be considered?	A complete case analysis was undertaken and results have been included in the manuscript as supplementary material Table S4. The text on p.10 lines 15-17 has been updated "A complete case analysis, which only included data from participant with complete HUQ and SF-36, was undertaken as a sensitivity analysis." And p.19 lines 18-19 and p.20 lines 1-5 "Sensitivity analyses are presented in Tables S4

	(complete case analysis) and S5 (analysis including participants who received the treatment they were randomised to). The conclusions remain the same in that septoplasty, is on average. more costly and more effective than medical management at 12-months. For both analyses the probability of septoplasty being considered cost-effective increases as the threshold for an additional QALY increases. However, septoplasty is unlikely to be considered cost-effective at 12-months at current thresholds for an additional QALY.”
"Similar to the primary outcome the economic analysis used data from the intention to-treat (ITT) population"<br>Can the authors please consider exploring a per protocol (PP) sensitivity anaysis?	Please see our response to editor above.
"The assumptions and estimates used in the economic analysis were subject to uncertainty hence stochastic sensitivity analyses using non-parametric bootstrapping were used. The bootstrapping technique using a SUR model allowed us to randomly select, with replacement, costs and effects simultaneously from individuals in each of the randomised groups. Differences between these pairs was then plotted on to the cost-effectiveness plane (19), this process was replicated for 1000 iterations. The results of the bootstrapping process were also combined with a variety of values for an additional QALY (£0 to £50k) to estimate the probability of each management strategy being considered cost-effective, these results were presented as cost effectiveness acceptability curves (CEACs)"<br>and<br>"Probabilistic sensitivity analysis (PSA) was undertaken to quantify any potential uncertainty in the model parameters as each model parameter, except the intervention costs (surgery and nasal spray) and transition probabilities for surgery which were fixed, had a measure of uncertainty surrounding it (SD) and was assigned a statistical distribution. The PSA facilitates the estimation of costs and effects using a set of parameters drawn from the statistical distribution using Monte Carlo simulations. Similarly to the bootstrapping of the within-trial results, 1000 Monte Carlo simulations were drawn to estimate the probability of septoplasty being considered cost-effective at a range of thresholds for an additional QALY."	Thank you for acknowledging the range of sensitivity analyses undertaken to explore potential uncertainty in our results.

Valuable additional analyses exploring uncertainty have been performed.	
"The trial findings were therefore extrapolated using a simple decision tree model in TreeAge (20) for an additional 12 months to estimate the cost-effectiveness of septoplasty compared with medical management at 24-months post-randomisation"<br>Can the outcomes of this extrapolation analysis, with uncertainty of the projection estimates quanitifed and communicated, please be shown in a Figure(s)?	Unfortunately, it is not possible to present this data in a Figure. Instead, we have provided the structure of the economic model, which extrapolated the trial outcomes, in Figure 1. We also presented the data used to populate the model, along with their measure of uncertainty and distribution in Table S3. Details of how these parameters were included in the model are described in detail on p.12 lines 15-25, p.13, and p.14 lines 1-3.
"Transition probabilities were based on the probability of septoplasty for each randomised arm "<br>and<br>"These assumptions were explored in sensitivity analyses which varied the transition probabilities +/- 50%. All model parameters are presented in Table S3. "<br>The authors do well to explore uncertainty in transition probabilities.	Thank you.
"Data on 307 participants (medical management (N=155), septoplasty (N=152)) from the ITT population were used in the economic analysis"<br>Can the authors please communicate the basis for the original sample size calculation?	The sample size calculation, which is based on the primary outcome (SNOT-22) is reported in the main trial paper (reference #5). The paper states “The sample of 378 participants was based on 90% power to detect a minimal clinically important difference of 9 points on SNOT-22,8 assuming a conservative standard deviation of 24 points15 and a type 1 error rate of 5%, and to allow for 20% drop-out.”
"Figure 2 illustrates the probably of septoplasty being considered cost-effective over a range of willingness to pay thresholds for an additional QALY"<br>Can the application of willingness to pay (WTP) please also be specified in the methods section?	This has been specified in the Methods section, please refer to p.11 lines 23-25 and p.12 lines 1-2.
"Table 3 Cost-effectiveness of septoplasty compared with medical management 13 at 24-months"<br>Can confidence intervals please be reported here?	Please see comment above.
Can the computation of ICER please be specified in the methods?	This has been stated on p.11 lines 13-15. “However, if an intervention was not dominant, then the incremental cost per additional QALY

	gained was estimated; this is done by dividing incremental costs by incremental QALYs (18).”
"Data to inform the micro-costing of septoplasty was only collected from those randomised to septoplasty hence variations in surgical costs could not be captured in the bootstrapping. • The 12-month trial follow-up, while pragmatic, was arguably too short for the full consideration of costs and benefits associated with a surgical intervention to be considered" [Strengths and Limitations of this Study]<br>and<br>"There are limitations associated with the economic analysis. Firstly, the micro costing estimates potentially underestimated the cost of surgery as additional costs such as overheads were not included and these costs were only sourced from one site. Additionally, data used to inform the micro-costing exercise was only collected for participants randomised to septoplasty, but 30% (n=47) of participants randomised to medical management underwent septoplasty. This has meant that variations in surgical costs were not captured in the bootstrapping analysis as every participant who underwent surgery was assigned the same cost. While pragmatically the trial had a 12-month follow-up, it was arguably too short a time horizon for costs and benefits associated with septoplasty to be considered. The economic model overcame this limitation however it should be noted that while validity checks and sensitivity analyses were undertaken to support the validity of the longer-term economic results, data to populate the model was taken from a single study and clinical advice"<br>The authors provide a discussion on the study limitations.	Thank you.

VERSION 2 - REVIEW

Reviewer **1**

Name **Youssef, Ahmed**

Affiliation **Alexandria Medical School, Otolaryngology/Head and Neck Surgery**

Date **01-Feb-2026**

COI

Good Study discussing the merits of surgery compared to medical treatment . I congratulate authors and totally agree with conclusions and outcomes.

**Reviewer**	**3**
**Name**	**Sunnergren, Ola**
**Affiliation**	**University of Gothenburg, Otorhinolaryngology**
**Date**	**07-Feb-2026**
**COI**	

I have read the revised manuscript and find it very well written. I have no further comments.

One minor typo: on line 11 in the Introduction, “underdoing” should be replaced with “undergoing”

**Reviewer**	**4**
**Name**	**Kelson, Zoe**
**Affiliation**	**University of Exeter, Mathematics**
**Date**	**27-Jan-2026**
**COI**	

Thanks to the authors for their responses.

Reviewer previous comment:

"Further details of the trial design, inclusion criteria and clinical effectiveness outcomes, including the primary outcome which was estimated at 6 months, are presented elsewhere "

Can the authors please provide a summary in brief here?

Author response:

"Please refer to reference #7 and #5."

Reviewer previous comment:

Can a copy of the original trial protocol please be provided?

Author response:

"The study protocol has been included as reference #7 and can be accessed https://pubmed.ncbi.nlm.nih.gov/3 2054508/"

Reviewer previous comment:

"All analyses were prespecified in a health economics analysis plan" Can this analysis plan please be provided?

Author response:

"Yes, the health economics plan can be shared."

Reviewer request:

Can the authors please further promote transparency by communicating a summary in brief of the study protocol and analysis plan in the main article?

Author responses:

"The study clinics recruited a diverse range of ethnicities and ages, as well as from both sexes, reflecting septoplasty operation provision in the NHS. The wide geographic spread of recruitment from both Teaching Hospitals and District General Hospitals as well as a range of different clinician graded surgeons performing the trial septoplasty ensured the trial participants were representative of the general UK population"

and

"explored the impact of the recruitment site on the costeffectiveness outcomes and can confirm it had no effect on costs (p= 0.083) or QALYs (p= 0.714). "

Reviewer request:

Can the above author responses please be incorporated into the article?

Author response:

"As reported in the main trial paper (see reference #5) randomisation was stratified by sex and baseline severity of symptoms according to scores on the NOSE scale: 30-50 (moderate), 55-75 (severe), 80- 100 (extreme)."

Reviewer request:

Can the methods for randomisation allocation please be clarified in the article?

Can stratification variables please be adjusted for in the analyses shown in this study?

Author response:

"For brevity overall response rates to questionnaires are not provided in this manuscript. The response rate for each individual variable at each time point is available online. Please see https://www.journalslibrary.nihr.ac.uk/ hta/MVFR4028 Appendix 2, Tables 74-76. The text on p.16 lines 6-7 have been updated to signpost the reader. “Response rates to each individual question at each timepoint are presented elsewhere(21).” "

Reviewer request:

Can the authors please supplement this description in the article by providing the range of proportions for missing data?

VERSION 2 - AUTHOR RESPONSE

Reviewer 4	
Can the authors please further promote transparency by communicating a summary in brief of the study protocol and analysis plan in the main article?	The text on p.7 lines 1-8 has been updated to the following to briefly summarise the study protocol “The NAIROS trial was a multicentre, non-adaptive, open label randomised controlled trial conducted across 17 otolaryngology clinics in England, Scotland and Wales. Participants were stratified by sex and baseline severity and were randomised 1:1 to the two management strategies. The primary outcome which estimated improvements in patient-reported outcomes was estimated at six-months using the Sino-Nasal Outcome Test (SNOT-22). Further details of the trial design, inclusion criteria and clinical effectiveness outcomes, including the primary outcome which was estimated at six-months, are presented elsewhere [5, 7].”<br><br>A summary of the economic analysis has already been provided on p6 lines 19-23 “The economic evaluation consisted of a within-trial analysis which estimated the incremental cost per quality-adjusted life year (QALY) gained at 12-months and a model-based analysis which estimated the incremental cost per QALY gained at 24-months. All analyses were prespecified in a health economics analysis plan.” The methods section of the manuscript also describes the analyses that were pre-specified in the Health Economics Analysis Plan.
Author responses:<br>"The study clinics recruited a diverse range of ethnicities and ages, as well as from both sexes, reflecting septoplasty operation provision in the NHS. The wide geographic spread of recruitment from both Teaching Hospitals and District General Hospitals as well as a range of different clinician graded surgeons performing the trial septoplasty ensured the trial participants were representative of the general UK population"<br>and<br>"explored the impact of the recruitment site on the cost-effectiveness outcomes and can confirm it	The Discussion section has been updated on p.24 lines 11-16 with the following “The study clinics recruited a diverse range of ethnicities and ages, as well as from both sexes, reflecting septoplasty operation provision in the NHS. The wide geographic spread of recruitment from both Teaching Hospitals and District General Hospitals as well as a range of different clinician graded surgeons performing the trial septoplasty ensured the trial

had no effect on costs (p= 0.083) or QALYs (p= 0.714). "<br>Reviewer request:<br>Can the above author responses please be incorporated into the article?	participants were representative of the general UK population."<br><br>The Results section has been updated on p.18 lines 8-9 with the following text "The potential impact of the recruitment site on the cost-effectiveness outcomes was confirmed and had no effect on costs (p= 0.083) or QALYs (p= 0.714)."
"As reported in the main trial paper (see reference #5) randomisation was stratified by sex and baseline severity of symptoms according to scores on the NOSE scale: 30-50 (moderate), 55-75 (severe), 80- 100 (extreme)."<br><br>Reviewer request:<br>Can the methods for randomisation allocation please be clarified in the article?<br>Can stratification variables please be adjusted for in the analyses shown in this study?	The text on p.7 lines 3-4 has been updated with the following "Participants were stratified by sex and baseline severity and were randomised 1:1 to the two management strategies."<br><br>As mentioned on p.11 lines 12-15 both gender and baseline severity of symptoms were adjusted for in the economic analysis. "Adjusted analyses, using seemingly-unrelated regression (SUR) and controlling for potential differences in age, gender, ethnicity, baseline SNOT-22, and baseline utility scores, was used to estimate incremental differences [17]."
"For brevity overall response rates to questionnaires are not provided in this manuscript. The response rate for each individual variable at each time point is available online. Please see https://www.journalslibrary.nihr.ac.uk/hta/MVFR4028 Appendix 2, Tables 74-76. The text on p.16 lines 6-7 have been updated to signpost the reader. "Response rates to each individual question at each timepoint are presented elsewhere(21)."<br><br>Reviewer request:<br>Can the authors please supplement this description in the article by providing the range of proportions for missing data?	The text on p.16 lines 7-8 has been updated with the following: "Missing responses to these questionnaires ranged from 2% at baseline to 30% at 12-months."
**Reviewer 1**	
Good Study discussing the merits of surgery compared to medical treatment . I congratulate authors and totally agree with conclusions and outcomes.	Thank you for your positive feedback.
**Reviewer 3:**	
I have read the revised manuscript and find it very well written. I have no further comments.<br>One minor typo: on line 11 in the Introduction,	Thank you for your positive feedback. The typo on line 11 in the Introduction has been corrected.

“underdoing” should be replaced with “undergoing”	

VERSION 3 - REVIEW

**Reviewer**	**4**
**Name**	**Kelson, Zoe**
**Affiliation**	**University of Exeter, Mathematics**
**Date**	**23-Apr-2026**
**COI**	

Thanks to the authors for their satisfactory responses and suitable revisions, which have improved clarity and strengthened the reporting of the study.
